# Expression and clinicopathological significance of human growth and transformation-dependent protein (HGTD-P) in uterine cervical cancer

**DOI:** 10.1111/j.1365-2559.2010.03627.x

**Published:** 2010-09

**Authors:** Young-Eun Cho, Jee-Youn Kim, Yong-Jun Kim, Youn-Wha Kim, Sun Lee, Jae-Hoon Park

**Affiliations:** Department of Pathology and Medical Research Center for Bioreaction to Reactive Oxygen Species, School of Medicine, Kyung Hee UniversitySeoul 130-701, Republic of Korea

*Sir*: Cervical cancer is the second most common malignancy in women worldwide. In developing countries, most cases are still diagnosed with locally advanced disease, so the great challenge in the management of advanced cervical cancer remains the control and prevention of distant metastasis. A prominent feature of clinically advanced cervical cancers is hypoxia^1^,^2^ which is, therefore, a therapeutic and prognostic factor associated with radio-resistance and worsened clinical outcome.^3^–^5^ A central coordinator of the hypoxic cellular response is hypoxia-inducible factor-1α (HIF-1α), a master transcription factor regulating the expression of numerous downstream targets.^6^,^7^ HIF-1α binding to hypoxia response elements at enhancers of target genes increases the expression of molecules which regulate angiogenesis, glucose transport, glycolysis, tissue invasion/metastasis and cell proliferation.^6^ Recently, we reported the identification of human growth and transformation-dependent protein (HGTD-P) as a new HIF-1α-responsive protein.^8^ Although the majority of genes downstream of HIF-1α participate in survival signalling pathways against hypoxic insult, increasing oxygen delivery or reducing metabolic demands for adaptation to hypoxic conditions,^9^–^11^ little is known about the role of HGTD-P during carcinogenesis.

**Figure 1:**
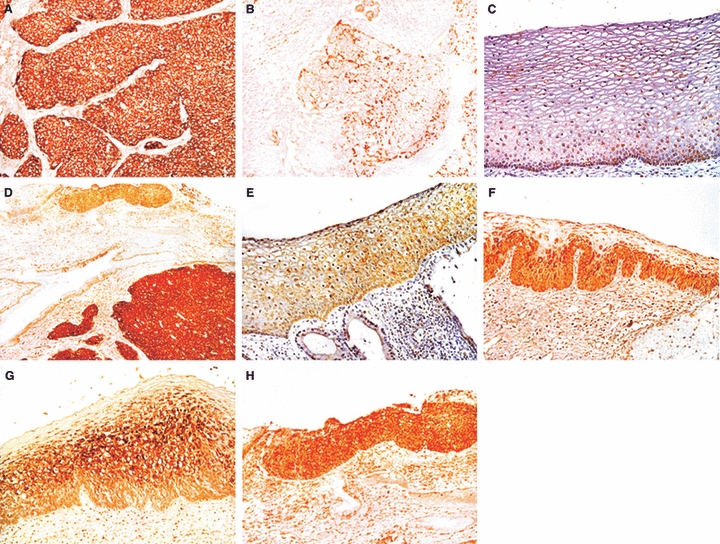
Immunohistochemical detection of human growth and transformation-dependent protein (HGTD-P) in cervical cancer (**A**, **B**), normal mucosa (**C**) and precancerous lesions (**E–H**). Representative samples of cervical cancer in which HGTD-P shows diffuse and strong staining in the cytoplasm of tumour cells (**A**), and no staining in tumour cells (**B**). **C**, Normal exocervical mucosa did not express HGTD-P. Among precancerous lesions including koilocytosis (**E**), cervical intraepithelial neoplasia (CIN)1 (**F**), CIN2 (**G**), CIN3 (**H**), HGTD-P expression was confined to dysplastic cells of the individual lesions. A representative case in which various cervical lesions are included within a microscopic field shows strong positive staining in cancer (c), less strong positive in CIN3 (ci) and no staining in normal cervical samples (n).

We investigated the expression of HGTD-P in human tissue samples of uterine cervical cancer (UCC; *n* = 85) and normal cervical tissue (*n* = 20) using immunohistochemistry with rabbit anti-HGTD-P antibody.^12^ As shown in Figure 1A, HGTD-P was stained strongly in the cytoplasm of the tumour cells, but no staining was observed in the nucleus or cytoplasmic membrane, whereas normal cervical mucosa showed no staining at all (Figure 1C). Most UCC tissues (70.6%) were positive for HGTD-P. Next, we aimed to determine the timing of aberrant HGTD-P expression during multistep cervical carcinogenesis and performed immunohistochemistry in cervical precancerous lesions, including cervical intraepithelial neoplasia (CIN)1 (*n* = 12), CIN2 (*n* = 7) and CIN3 (*n* = 13). As a result, fewer than half the precancerous lesions were stained moderately with HGTD-P, which was confined to dysplastic cells (Figure 1E–H). In fact, 33.3% of CIN1, 28.6% of CIN2 and 46.2% of CIN3 expressed cytoplasmic HGTD-P, whereas 70.6% of UCC was positive for HGTD-P (Table 1). The cases in which UCC tissue samples included normal cervical mucosa and precancerous lesions on a slide demonstrated diverse patterns of HGTD-P expression that were dependent upon the type of cervical lesion (Figure 1D). For example, HGTD-P was stained strongly in UCC and moderately in CIN3, whereas normal mucosa showed no staining. These results suggest that HGTD-P expression is a common and cancer-specific event in the uterine cervix, occurring at an early stage of cervical carcinogenesis.

**Table 1:** Comparison of human growth and transformation-dependent protein (HGTD-P) expression in cervical cancer and precancerous lesions

HGTD-P expression	CIN1	CIN2	CIN3	Cervical cancer	Total
Negative	8 (66.7)	5 (71.4)	7 (53.8)	25 (29.4)	45

Positive	4 (33.3)	2 (28.6)	6 (46.2)	60 (70.6)	72

Total	12	7	13	85	117

CIN, cervical intraepithelial neoplasia.

*P* = 0.009, analysed by Pearson's chi-square test.

**Figure 2:**
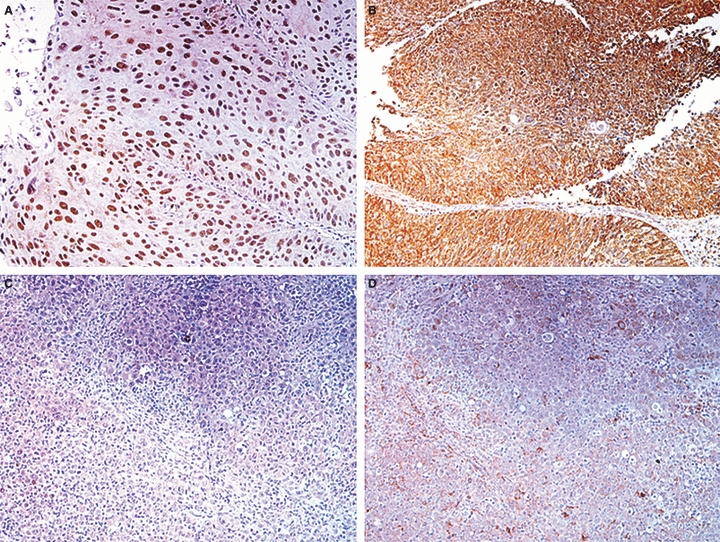
Immunohistochemistry of hypoxia-inducible factor-1α (HIF-1α) (**A**, **C**) and human growth and transformation-dependent protein (HGTD-P) (**B**, **D**) in identical tumour samples. **A**, **B**, Tumour cells strongly expressed both HIF-1 and HGTD-P. **C**, **D**, The tumour expressed neither HIF-1α nor HGTD-P.

To determine the association between HGTD-P and HIF-1α in human clinical samples, we performed immunohistochemistry for HIF-1α in UCC tissues (*n* = 15) and compared the result of HIF-1α staining to that of HGTD-P expression. As a result, HIF-1α overexpression was detected in 60% of UCC tissues; 77.8% of HIF-1α-overexpressed tumours also expressed HGTD-P. The correlation of expression between these two proteins was statistically significant (Table 2; *P* = 0.04). Representative cases of UCC with positive correlation between the two proteins are shown in Figure 2. This finding suggests that HGTD-P participates in a molecular mechanism of hypoxia-regulated tumour development in association with HIF-1α, and furthermore is of great value for future investigation regarding its role in cervical carcinogenesis.

**Table 2:** Comparison of human growth and transformation-dependent protein (HGTD-P) expression with hypoxia-inducible factor-1α (HIF-1α) expression in uterine cervical cancer

	HIF-1α expression (%)		
	
HGTD-P expression	Negative	Positive	Total
Negative	5 (83.3)	2 (22.2)	7

Positive	1 (16.7)	7 (77.8)	8

*P* = 0.042, analysed by Fisher's exact test.

**Figure 3:**
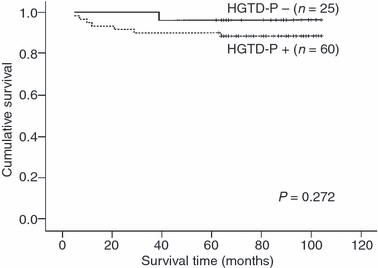
Overall survival curves in patients with uterine cervical cancer depending upon human growth and transformation-dependent protein (HGTD-P) expression. The patients with HGTD-P-expressing tumours tend to have poor overall survival rate, compared to those with tumours that do not express HGTD-P.

Next, we determined any clinicopathological significance of the aberrant expression of HGTD-P in UCC and compared HGTD-P-positive tumours to -negative tumours in relation to clinicopathological parameters. HGTD-P-positive-tumours tend to be associated with advanced International Federation of Gynecology and Obstetrics (FIGO) stage, parametrial invasion and tumour recurrence, but this association did not reach statistical significance (Table 3). The overall survival rate was poorer in patients with HGTD-P expression than in those without HGTD-P expression (Figure 3). However, this difference did not reach statistical significance (*P* = 0.272).

**Table 3:** Correlation of human growth and transformation-dependent protein (HGTD-P) expression in uterine cervical cancer with clinicopathological factors

		HGTD-P expression		
			
Factors	No.	−(*n* = 25)	+(*n* = 60)	*P*-value*
Age (years)				
≤45	35	11	24	0.733
	
>45	50	14	36	

Histological type				
Squamous cell carcinoma	63	16	47	0.355
	
Adenocarcinoma	15	7	8	
	
Others	7	2	5	

FIGO stage				
IA1-IB1	61	21	40	0.122
	
IB2-IIIB	24	4	20	

LN metastasis				
No	71	20	51	0.760
	
Yes	12	4	8	

Parametrial invasion				
No	24	24	47	0.056
	
Yes	1	1	13	

Death				
No	75			

Yes	10			

FIGO, International Federation of Gynecology and Obstetrics; LN, lymph node.

* Fisher's exact test.

In summary, we have shown that HGTD-P expression is a frequent event in UCC and is associated significantly with HIF-1α expression in human cervical cancer samples. During multistep cervical carcinogenesis, the aberrant expression of HGTD-P protein occurs in CIN1 and might accumulate in the process of tumorigenesis. Moreover, HGTD-P expression is correlated with parametrial invasion. These facts suggest that HGTD-P plays an important role in cervical carcinogenesis. Moreover, HGTD-P can be considered a valuable tumour marker of cervical neoplasia. However, the molecular mechanism of HGTD-P involvement in tumour development and progression in UCC remains to be elucidated.
